# Multicenter Clinical Evaluation of ETEST Eravacycline for Susceptibility Testing of *Enterobacteriaceae* and Enterococci

**DOI:** 10.1128/jcm.01650-22

**Published:** 2023-03-06

**Authors:** Laurine S. Blanchard, Thomas P. Armstrong, Michael Kresken, Christopher L. Emery, Yun X. Ying, Véronique Sauvonnet, Gilles Zambardi

**Affiliations:** a bioMérieux, SA, Marcy l’Etoile, France; b bioMérieux Inc., Hazelwood, Missouri, USA; c Antiinfectives Intelligence, GmbH, Cologne, Germany; d Indiana University School of Medicine, Indianapolis, Indiana, USA; e Quest Diagnostics, Lewisville, Texas, USA; f bioMérieux, SA, La Balme-les-Grottes, France; NorthShore University HealthSystem

**Keywords:** antimicrobial susceptibility testing, ETEST, *Enterobacteriaceae*, *Enterococcus* spp., eravacycline, gradient methods

## Abstract

Eravacycline (ERV) (brand name Xerava [Tetraphase]) is a new tetracycline-class antibacterial that has been approved by the U.S. Food and Drug Administration (FDA) and the European Medicines Agency (EMA) for treatment of complicated intra-abdominal infections (cIAIs). ETEST is a gradient diffusion method that represents a simple alternative to the broth microdilution (BMD) method for performing antimicrobial susceptibility testing (AST). A multicenter evaluation of the performance of the new ETEST ERV (bioMérieux) in comparison with BMD was conducted following FDA and International Standards Organization (ISO) recommendations, using FDA- and EUCAST-defined breakpoints. Clinical isolates of *Enterobacteriaceae* (*n* = 542) and *Enterococcus* spp. (*n* = 137) were included. Based on the BMD reference method, 92 *Enterobacteriaceae* isolates and 9 enterococcal isolates were nonsusceptible to ERV according to the FDA breakpoints, while 7 Escherichia coli isolates and 3 *Enterococcus* sp. isolates were classified as ERV resistant according the EUCAST breakpoints. Referring to FDA performance criteria, the ETEST ERV demonstrated 99.4% and 100.0% essential agreement (EA), 98.0% and 94.9% categorical agreement (CA), very major error (VME) rates of 5.4% and 33.33%, and major error (ME) rates of 1.3% and 3.1% with clinical and challenge isolates, respectively, of *Enterobacteriaceae* and *Enterococcus* spp. According to EUCAST breakpoints, E. coli and *Enterococcus* sp. isolate results also met ISO acceptance criteria for EA and CA (EA of 99.0% and 100.0%, respectively, and CA of 100.0% for both), without any VMEs or MEs. In conclusion, we report that ETEST ERV represents an accurate tool for performing ERV AST of *Enterobacteriaceae* and *Enterococcus* sp. isolates.

## INTRODUCTION

Eravacycline (ERV) was the first fully synthetic tetracycline approved by the U.S. Food and Drug Administration (FDA) and the European Medicines Agency (EMA) in 2018 for treatment of complicated intra-abdominal infections (cIAIs) in adults. Like other drugs from the tetracycline family, ERV inhibits bacterial protein synthesis by binding to the 30S ribosomal subunit. ERV is structurally similar to tigecycline except that ERV has a fluorine atom at the C-7 position and a pyrrolidinoacetamido group at the C-9 position; these changes allow this novel, synthetic fluorocycline to retain activity against bacteria expressing efflux pumps and ribosomal protective proteins, which are the two most common tetracycline resistance phenotypes (1). The spectrum of ERV activity includes anaerobes, Gram-positive organisms, including vancomycin-resistant enterococci (VRE) and methicillin-resistant Staphylococcus aureus (MRSA), and Gram-negative bacteria, including strains producing notably extended-spectrum β-lactamases (ESBLs) and carbapenem-resistant *Enterobacterales* (CRE) (2, 3). Recently, ERV activity was retained against multidrug-resistant (MDR) isolates, including many carbapenem-resistant Escherichia coli strains, such as carbapenemase-producing E. coli strains, and other Gram-negative pathogens expressing KPC and OXA carbapenemases but also VRE (4–6). Thus, ERV is a promising carbapenem-sparing alternative for treatment of extended-spectrum-cephalosporin-resistant E. coli infections and an option for treatment of carbapenem-resistant Klebsiella pneumoniae invasive infections (4, 7). Combined with polymyxin B, ERV has synergistic effects against clinically common carbapenem-resistant E. coli and K. pneumoniae strains (8). ERV demonstrated a high level of clinical efficacy and a good safety and tolerability profile, with a broad spectrum of activity, flexibility for use in patients with renal injury or antibiotic allergies, and positive clinical outcomes in a large real-world cohort (9, 10). Fewer adverse events have been observed with ERV than with tigecycline (10).

Resistance to ERV is associated with upregulated, nonspecific intrinsic MDR efflux and certain target site modifications (2). Emergence of resistance among enterococci is also possible due to a combination of *rpsJ* mutations and horizontal transfer of* tet* genes (5). Indeed, the emergence of resistance to promising agents such as ERV and plazomicin highlights the need for continuous surveillance and application of enhanced antimicrobial stewardship (11).

This study evaluated the performance of ETEST ERV (bioMérieux, Marcy-l’Étoile, France), a new gradient diffusion strip (CE marked and FDA cleared [12]), for determining the MICs for some *Enterobacteriaceae* species, including Citrobacter freundii, Citrobacter koseri, Enterobacter cloacae, Escherichia coli, Klebsiella oxytoca, Klebsiella aerogenes, Klebsiella pneumoniae, Enterococcus faecalis, and Enterococcus faecium, compared to the Clinical and Laboratory Standards Institute (CLSI)/ International Standards Organization (ISO) 20776-1 broth microdilution (BMD) reference method (13–16). ETEST ERV is neither FDA cleared nor CE marked for use for Staphylococcus aureus, Streptococcus viridans group strains, or anaerobic organisms.

## MATERIALS AND METHODS

### Participating institutions and ethical clearance.

The study was conducted at four different sites, including two in the United States, namely, Indiana University School of Medicine (IUSM) (Indianapolis, IN, USA) and Quest Diagnostics (Quest) (Lewisville, TX, USA). and two in Europe, namely, Antiinfectives Intelligence, GmbH (AI) (Cologne, Germany), and bioMérieux SA (Marcy) (Marcy-l’Etoile, France). Each U.S. study site performing testing on clinical strains acquired local institutional review board approval or waiver thereof prior to study initiation.

### Clinical bacterial isolates.

In the clinical study, 600 clinical isolates were tested at three sites (200 at AI, 199 at Quest, and 201 at IUSM). Among the 600 clinical isolates tested, 390 belonged to the following indicated species approved by the EMA: E. coli, K. pneumoniae, Enterococcus faecalis, and E. faecium. Clinical isolates were acquired from routine cultures processed in the clinical laboratory at each trial site. The overall distribution of these clinical isolates according to source of infection was as follows: urine, 43.00%; skin and soft tissue, 18.67%; blood and cerebrospinal fluid, 14.67%; respiratory tract, 10.67%; body fluid, 9.0%; digestive tract, 2.83%; orthorhinolaryngeal, 0.67%; others, 0.5% (3 isolates). Clinical isolates were identified to the genus and species level using a matrix-assisted laser desorption ionization–time of flight mass spectrometry (MALDI-TOF MS) technique. Technicians performing the clinical trial testing did not have prior knowledge of any contemporary clinical isolate’s susceptibility results. Duplicate isolates from the same patient were excluded from the clinical trial. Of the 600 clinical isolates, 469 (78.2%) were contemporary isolates (tested within 6 months after isolation in culture, not preselected, and, if frozen, minimally subcultured) and 131 (21.8%) were stock (frozen with no time constraints, minimally subcultured, and occasionally favoring the inclusion of resistant strains). Among clinical isolates, 78/600 isolates (13.0%) were nonsusceptible to ERV by BMD according to FDA interpretive criteria (susceptible for *Enterobacteriaceae*, ≤0.5 μg/mL; susceptible for Enterococcus faecium and E. faecalis, ≤0.064 μg/mL). Using EUCAST breakpoints, 8/300 (2.7%) of the clinical isolates were resistant to ERV by the reference method (for Escherichia coli: susceptible, ≤0.5 μg/mL; resistant, >0.5 μg/mL; for *Enterococcus* spp., susceptible, ≤0.125 μg/mL; resistant, >0.125 μg/mL).

### Characterization of ERV challenge set isolates.

The Marcy site tested 79 challenge isolates, including 62 *Enterobacteriaceae* isolates and 17 Enterococcus faecalis or E. faecium isolates (the composition of the challenge set is presented in Table 2). There were 23 ERV-nonsusceptible isolates by BMD according to FDA breakpoints and 6 VRE strains, including 5 E. faecalis strains harboring *vanA* and 1 E. faecium strain harboring *vanB*. Resistance phenotypes of the challenge set isolates with tetracycline, glycopeptides, and β-lactams were determined using the Vitek 2 system. The resistance mechanisms were identified by whole-genome sequencing. Raw data were obtained after DNA extraction with the DNeasy UltraClean microbial kit (Qiagen, Hilden, Germany) and sequencing (paired-end 2 × 150-bp reads) on the Illumina (San Diego, the USA) platform. The antimicrobial susceptibility testing (AST) analysis was performed in three steps, as follows: (i) *de novo* assembly of Illumina reads (IDBA-UD assembler), (ii) search for resistance genes with BLASTn versus an internal nucleic acid base including the prevalent acquired genes for the studied antibiotic families (β-lactamases, *van*, and *tet*), and (iii) identification of the alleles of certain β-lactamase genes (*tem*, *oxa-48 like*, and *shv*) by a BLASTx search versus an internal protein base. E. coli challenge strains expressed a variety of resistance mechanisms, such as acquired β-lactamases, including ESBLs and carbapenemases. Among the 33 tetracycline-resistant strains, there were 20 *Enterobacteriaceae* strains, including 13 harboring *tet*(A), 2 *tet*(B), 1 *tet*(B) plus *tet*(M), and 4 *tet*(D). Among the 13 *Enterococcus* sp. strains, 1 E. faecalis strain harbored *tet*(D), *tet*(S), and truncated *tet*(M), 10 *Enterococcus* sp. strains harbored *tet*(M), and 2 E. faecalis strains harbored *tet*(S).

### Study setting and design.

The performance of the ETEST ERV was compared to that of the BMD reference method following CLSI M07-Ed11 (15) and ISO 20776-1 (16) standards. The study design included four performance components, i.e., (i) a challenge study, (ii) a clinical study, (iii) a quality control (QC) study, and (iv) a reproducibility study (17, 18). These four substudies included isolates of the following species: Citrobacter freundii, Citrobacter koseri, Enterobacter cloacae, Escherichia coli, Klebsiella aerogenes, Klebsiella oxytoca, Klebsiella pneumoniae, Enterococcus faecalis, and Enterococcus faecium. Inoculum purity was checked for all isolates. Inoculum density was verified by colony count for all QC replicates, all reproducibility tests, and 10% of the contemporary clinical isolates, following FDA guidance (17). Challenge, reproducibility, and QC studies took place at Marcy, while clinical, reproducibility, and QC studies took place at IUSM and Quest. AI performed clinical and QC studies.

### Susceptibility testing methodology.

**FIG 1:**
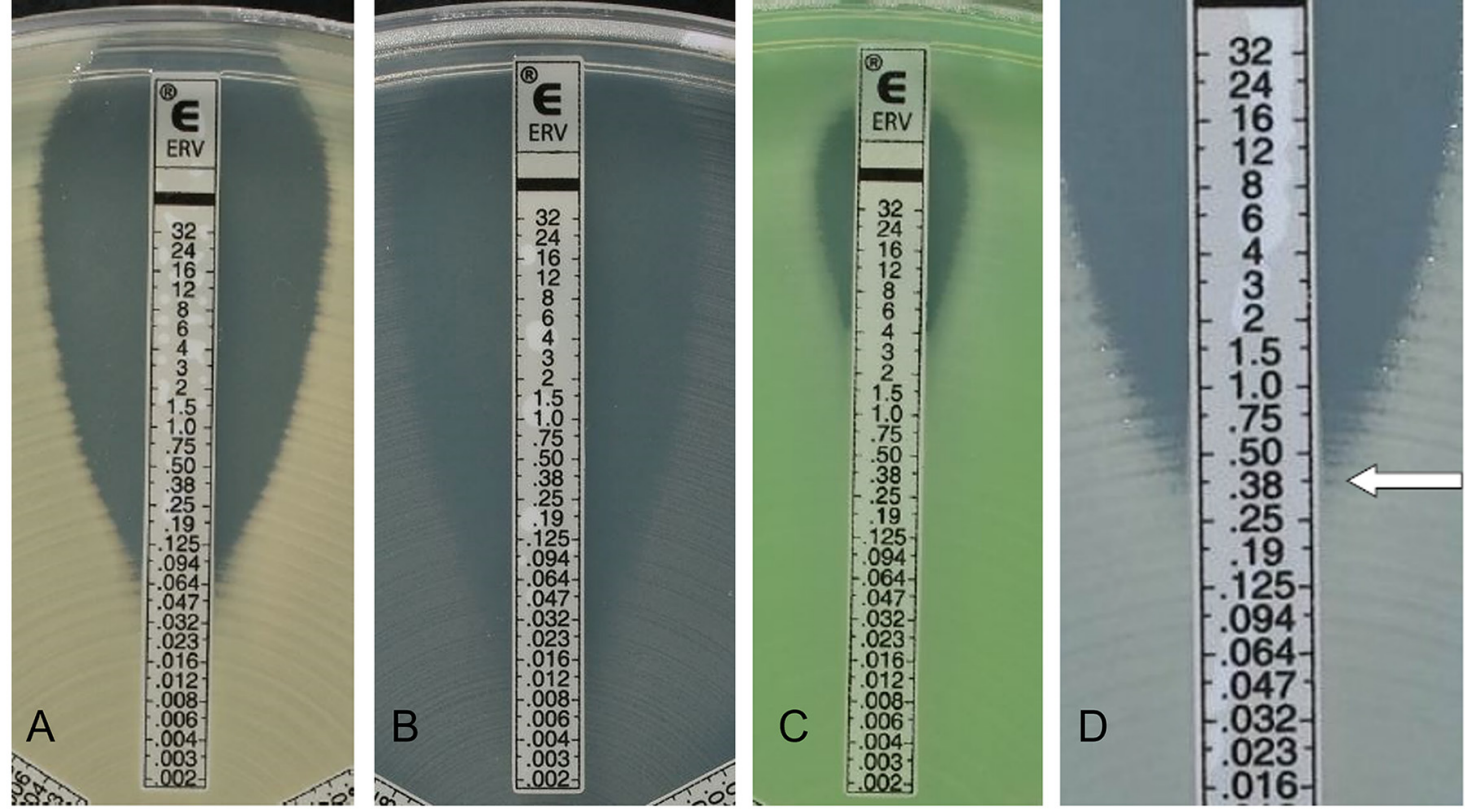
Examples of readings with ETEST ERV. Examples of tested organisms and their MIC readings are shown. (A) E. coli ATCC 25922 (MIC = 0.064 μg/mL). (B) E. faecalis ATCC 29212 (MIC = 0.023 μg/mL). (C) P. aeruginosa ATCC 27853 (MIC = 4 μg/mL). (D) Enterobacter cloacae isolate (MIC = 0.38 μg/mL); for bacteriostatic agents, results are read at 80% inhibition of growth.

A visual calibrator was used to prepare a 0.5 McFarland inoculum (for nonmucoid isolates) in 0.85% sterile saline from 18- to 24-h growth on tryptic soy or Columbia agar plates supplemented with 5% sheep blood. For mucoid isolates, a 1.0 McFarland standard inoculum was prepared for ETEST testing and a 0.5 McFarland suspension was prepared for BMD testing. Within 15 min after preparation, a sterile cotton swab moistened with the standardized bacterial suspension was inoculated manually or automatically using the Retro C80 rota-plater on BBL Mueller-Hinton II agar plates (BD, Sparks, MD), and ETEST strips were applied to the plates with an applicator (Nema C88 vacuum pen; bioMérieux; Durham, NC) or forceps. Different lots of Mueller-Hinton agar plates from the same manufacturer (BD) were used. Plates were incubated in ambient air at 35 ± 2°C, and results were read after 16 to 20 h of incubation. In case of trailing growth, the MIC was read at the concentration of ERV showing 80% inhibition of growth (the point at which significant inhibition of bacterial growth intersected the ETEST ERV strip, as judged by the naked eye [bacteriostatic reading]), as described by the ETEST ERV instructions for use (bioMérieux). An example of MIC determination is shown in Fig. 1. MICs falling between two dilutions were rounded up to the next highest value. Nondoubling MIC values (e.g., 0.75 and 3) were rounded up, if necessary, to the standard doubling dilution before categorization (Fig. 1) and comparison to the reference method. BMD testing was performed using 96-well plates prepared at the bioMérieux facilities (La Balme-les-Grottes, France) in compliance with the directions in CLSI M07-Ed11 (15) and ISO 20776-1 (16) standards. The BMD panels consisted of 2-fold dilutions of ERV in cation-adjusted Mueller-Hinton broth. Prepared panels were concentrated twice to reach final concentrations after inoculation ranging from 0.002 to 32 μg/mL. Each batch produced was controlled by inoculating several panels selected at the beginning, at the middle, and at the end of production with the QC strains recommended by the CLSI M100 standard (14). The panels were then frozen at −80°C and shipped in aluminum pouches on dry ice, with constant monitoring of the temperature during transportation, to all clinical trial sites. Prior to use, BMD panels were completely thawed at room temperature for 30 min to 1 h. Using a repeating pipette, BMD panels were inoculated with 50 μL per well of a 100-fold dilution of the original bacterial suspension in BBL cation-adjusted Mueller-Hinton II broth (BD) of the same 0.5 McFarland suspension used for ETEST ERV and incubated at 35 ± 2°C in ambient air for 16 to 20 h. Results were read after 16 to 20 h of incubation. For some species, in testing of tetracycline antibiotics like ERV by BMD, trailing growth can make endpoint determination difficult, as described previously (14). In such cases, the MIC should be read at the lowest concentration at which the trailing begins, and tiny buttons should be ignored (see Fig. 3 and 4 in reference 15). An aliquot was removed from each growth control well of the BMD panels, inoculated on blood agar, and assessed for purity after 20 to 24 h and 44 to 48 h of incubation. Inoculum density checks were performed by plating 100 μL of a 1:1,000 dilution of the growth control from BMD panels onto a blood agar plate, which was subsequently incubated at 35 ± 2°C in ambient air for 18 to 48 h. After incubation, colony counts were recorded and used to calculate inoculum density.

### Reproducibility study.

Ten on-scale isolates provided by bioMérieux (C. freundii, *n* = 1; E. cloacae, *n* = 1; E. coli, *n* = 3; K. pneumoniae, *n* = 1; K. oxytoca, *n* = 1; E. faecium, *n* = 1; E. faecalis, *n* = 2) were tested at the IUSM, Quest, and Marcy study sites. Isolates were subcultured twice on blood agar before testing. Each isolate was tested in triplicate on 3 different days. Three separate 0.5 McFarland suspensions of each isolate were prepared in normal saline for the ETEST and inoculum density check. Different lots of Mueller-Hinton plates from the same manufacturer (BD) were used. The results from all three sites were used to compute a modal MIC value for each strain used in the reproducibility study (if there was no modal value, then the median was used). The total number of results within one doubling dilution of the mode or median was used to calculate the reproducibility rate as the percentage of the total number of tests. Best case calculations for reproducibility assumed that off-scale values were within one doubling dilution of the mode. Worst case reproducibility assumed that off-scale values were not within one doubling dilution. Performance was evaluated using FDA and ISO performance criteria, i.e., ≥95% reproducibility.

### QC study.

Three ATCC reference strains for ETEST ERV and four ATCC reference strains for BMD, as recommended by CLSI M100-Ed29 (14) and the EUCAST v9.0 QC table (19), were tested as QCs on each day of comparative testing. Each isolate was tested a minimum of 20 times at each site. QC strains included the following organisms: Escherichia coli ATCC 25922 (CLSI/EUCAST range, 0.032 to 0.125 μg/mL), Enterococcus faecalis ATCC 29212 (CLSI/EUCAST range, 0.016 to 0.064 μg/mL), and Pseudomonas aeruginosa ATCC 27853 (CLSI range, 2 to 16 μg/mL). Staphylococcus aureus ATCC 29213 was used solely for BMD (CLSI/EUCAST range, 0.016 to 0.125 μg/mL). QC strains were subcultured twice on blood agar before testing. QC ranges described by CLSI M100 Ed-29 were verified on each day of clinical or challenge testing, and an inoculum density check was conducted for all QC tests according to CLSI M07-Ed11 guidelines (14, 15). Results were considered invalid if QC results were out of range. QC performance for ETEST ERV was calculated as the percentage of results within the expected range. Performance was evaluated using FDA and ISO performance criteria, i.e., QC results within the expected range ≥95% of the time.

### Clinical and challenge studies.

Clinical and challenge isolates were evaluated for ERV susceptibility using ETEST ERV and reference BMD simultaneously using the methods described above. Every isolate was subcultured twice on blood agar before testing.

### Data analysis.

Clinical and challenge data were combined in the performance evaluation. MIC values were interpreted using FDA and EUCAST breakpoints (13, 20). Essential agreement (EA) was defined as the percentage of total isolates for which the test and reference methods were within one doubling dilution of each other. Category agreement (CA) was defined as the percentage of total test results within the interpretive category (susceptible, intermediate, and resistant [EUCAST] or nonsusceptible [FDA]) with agreement with the reference method using FDA interpretative criteria as indicated. The very major error (VME) rate was defined as the percentage of isolates interpreted as resistant or nonsusceptible by the reference method that were susceptible by the test method. The major error (ME) rate was defined as the percentage of isolates interpreted as susceptible by the reference method that were resistant or nonsusceptible by the test method. In accordance with the FDA response to the Susceptibility Testing Manufacturers Association (STMA) letter dated 3 November 2015, for drugs for which there is no intermediate breakpoint and for which the VME rate and/or the ME rate is unacceptable according to the FDA performance criteria, the VME rate and/or the ME rate may be adjusted to exclude the VME results and/or the ME results within EA (21). Test MIC values from challenge and clinical results in the frequency table were separated into three categories, i.e., (i) values at least one dilution lower than the reference method MIC values, (ii) values equal to the reference method MIC values, and (iii) values at least one dilution higher than the reference method MIC values. Trending is the evaluation of test device results to determine whether the results that differ from the reference method are significantly skewed or predominantly in one direction. Trending may be used to compare results between different susceptibility testing methods to assess bias that would not be evident using EA or CA unless larger numbers of organisms were evaluated. A trend of ≥30% must be reflected in the labeling. Performance was evaluated using FDA and ISO performance criteria, as follows: EA and CA, ≥90%; ME rate, ≤ 3.0%; VME rate, ≤ 2.0% (FDA) or ≤3% (ISO); trending,  <30% (17, 18, 22).

## RESULTS

### QC of ETEST ERV.

To perform the QC of the ETEST ERV, three QC organisms were tested a minimum of 20 times with ETEST at each site and four were tested a minimum of 20 times with the BMD method throughout the study at all study sites, as described above. One hundred percent of BMD and ETEST results for E. faecalis ATCC 29212 were within the expected range defined by the CLSI M100 standard (14). BMD results for S. aureus ATCC 29213 were out of range once, resulting in 98.8% of all QC results (80/81 results) being within the required range. All reference BMD QC results for Escherichia coli ATCC 25922 were within range (81/81 results [100%]), whereas 2 were out of range with ETEST, leading to 97.5% of results (79/81 results) being within range. For P. aeruginosa ATCC 27853, 98.8% of BMD and ETEST results (80/81 results) were within the expected range, thus meeting both FDA and ISO requirements.

### Reproducibility of ETEST ERV.

Evaluation in triplicate for 3 days resulted in 90 results per site, with 270 results in total. Mode values of ERV MICs for each isolate tested across all sites are shown in Table 1. The rate of reproducibility between sites for all strains (268/270 results) was 99.3% within one doubling dilution of strain-specific modal values. Two sites (IUSM and Quest) showed within-site reproducibility rates of 100% (90/90 results), while the Marcy rate was 97.8% (88/90 results).

**TABLE 1:** Reproducibility performance of ETEST ERV

Organism	MIC test mode (μg/mL)	No. of doubling dilutions from mode:						
		Off-scale	−2	−1	0	+1	+2	Off-scale
Citrobacter freundii 0810174	0.25	0	0	0	27	0	0	0
Enterobacter cloacae 1609167	4	0	0	0	25	2	0	0
Enterococcus faecalis 1302326	0.064	0	0	9	18	0	0	0
Enterococcus faecalis 9710048	0.032	0	0	0	19	8	0	0
Enterococcus faecium 1503019	0.125	0	0	0	13	12	2	0
Escherichia coli 1012108	0.5	0	0	0	27	0	0	0
Escherichia coli 1703098	0.25	0	0	0	27	0	0	0
Escherichia coli 1606403	0.25	0	0	0	22	5	0	0
Klebsiella oxytoca 0503045	1	0	0	0	14	13	0	0
Klebsiella pneumoniae 1606357	0.5	0	0	0	27	0	0	0
Total		0	0	9	219	40	2	0

### Challenge and clinical performance.

Evaluation of the performance of the ETEST ERV included data for 679 isolates from the challenge and clinical studies combined.

### (i) ETEST ERV performance with FDA breakpoints and FDA requirements for *Enterobacteriaceae* and *Enterococcus* isolates.

**FIG 2:**
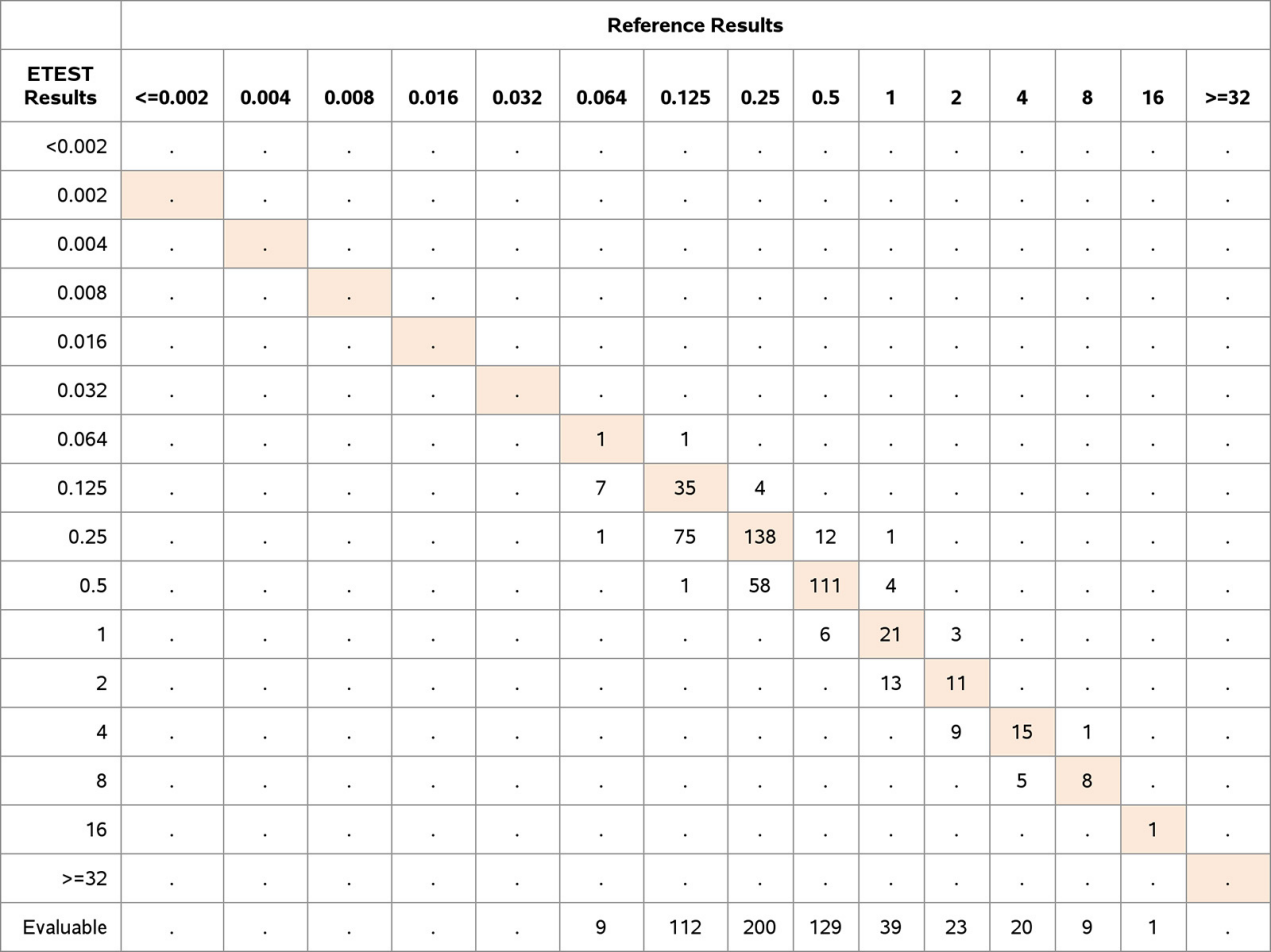
Distribution of MICs (in micrograms per milliliter) determined by ETEST and BMD for *Enterobacteriaceae*. Clinical and challenge isolates of *Enterobacteriaceae* (*n* = 542) were tested for ERV susceptibility with ETEST ERV and the BMD reference method. The numbers of isolates with exact MIC agreement between the ETEST and BMD results are shaded in orange.

**FIG 3:**
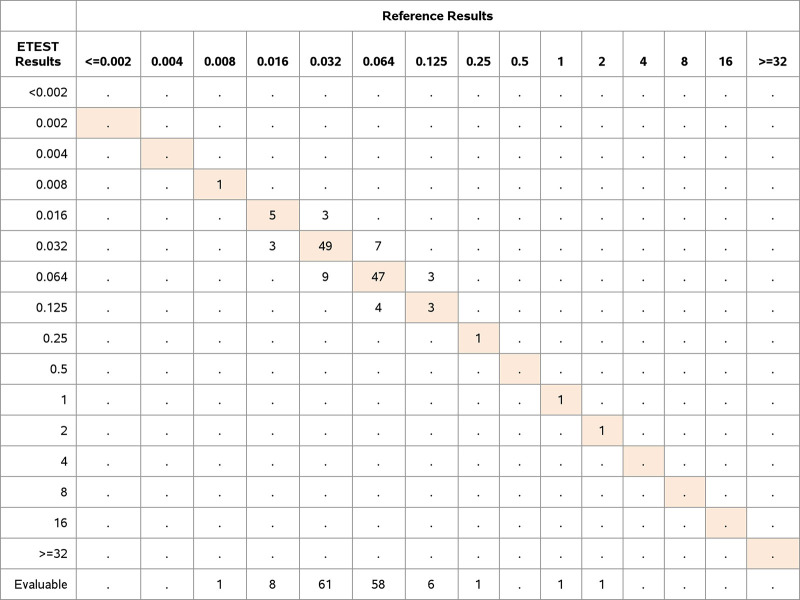
Distribution of MICs (in micrograms per milliliter) determined by ETEST and BMD for *Enterococcus* spp. Clinical and challenge isolates of *Enterococcus* spp. (*n* = 137) were tested for ERV susceptibility with ETEST ERV and the BMD reference method. The numbers of isolates with exact MIC agreement between the ETEST and BMD results are shaded in orange.

A total of 101 *Enterobacteriaceae* and *Enterococcus* isolates (14.9%) were nonsusceptible to ERV by BMD according to FDA breakpoints. ETEST ERV demonstrated 99.6% EA (676/679 isolates), 97.3% CA (661/679 isolates), a VME rate of 7.9% (8/101 isolates), and an ME rate of 1.7% (10/578 isolates). Seven of the VMEs were within EA. In accordance with the FDA response to the STMA letter dated 3 November 2015, for drugs for which there is no intermediate breakpoint and for which the VME or ME rate is unacceptable according to the FDA performance criteria, the VME rate was adjusted to exclude the VME results that were within EA. The adjusted VME rate was 1.0% (1/101 isolates) (Table 2). The distribution of species, nonsusceptibility to ERV, and performance determined using FDA breakpoints are shown in Fig. 2, Fig. 3, and Table 2. All species tested demonstrated CA of >90%.

**TABLE 2:** Clinical and challenge performance of ETEST ERV with FDA breakpoints

Organism	Total no.	No. within EA	EA (%)	Total no. evaluable	No. evaluable within EA	No. within CA	CA (%)	No. nonsusceptible	No. of VMEs^*a*^	No. of MEs
Challenge set										
C. freundii	10	10	100.0	10	10	8	80.0	4	0	2
E. cloacae	12	12	100.0	12	12	12	100.0	6	0	0
E. faecalis	14	14	100.0	14	14	14	100.0	1	0	0
E. faecium	3	3	100.0	3	3	3	100.0	0	NA	0
E. coli	11	11	100.0	11	11	11	100.0	1	0	0
K. aerogenes	3	3	100.0	3	3	3	100.0	1	0	0
K. oxytoca	12	12	100.0	12	12	12	100.0	0	NA	0
K. pneumoniae	14	14	100.0	14	14	13	92.9	10	1	0
Subtotal	79	79	100.0	79	79	76	96.2	23	1	2
Clinical set										
C. freundii	60	60	100.0	60	60	58	96.7	9	0	2
C. koseri	30	30	100.0	30	30	30	100.0	0	NA	0
E. cloacae	60	60	100.0	60	60	56	93.3	20	3	1
E. faecalis	60	60	100.0	60	60	54	90.0	4	2	4
E. faecium	60	60	100.0	60	60	59	98.3	4	1	0
E. coli	180	178	98.9	180	178	180	100.0	6	0	0
K. aerogenes	29	29	100.0	29	29	29	100.0	5	0	0
K. oxytoca	31	31	100.0	31	31	31	100.0	6	0	0
K. pneumoniae	90	89	98.9	90	89	88	97.8	24	1	1
Subtotal	600	597	99.5	600	597	585	97.5	78	7	8
Combined set, total	679	676	99.6	679	676	661	97.3	101	8^*b*^	10

a NA, not applicable.

b One VME after adjustment (21).

The proportion of clinical isolates that were nonsusceptible to ERV as determined by ETEST and BMD for *Enterobacteriaceae* was 14.6% according to the FDA breakpoints. Overall performance characteristics of the ETEST ERV with all clinical and challenge isolates from the species we tested in the *Enterobacteriaceae* family were 99.4% EA, 98.0% CA, VME rate of 5.4% but adjusted VME rate of 1.1%, and ME rate of 1.3% (Fig. 2 and Table 2). After adjustment, 1 VME was observed for a K. pneumoniae isolate; the ETEST ERV MIC was 0.25 μg/mL, and the BMD MIC was 1 μg/mL. This isolate was not evaluated for any resistance mechanism. Upon repeat testing in triplicate, all results were in agreement (susceptible with both methods in every test). The FDA does not allow categorical error resolution. The initial results were retained for the FDA analysis. The adjusted VME rate was 2.9% for K. pneumoniae (1/34 isolates). Nevertheless, the ETEST ERV met the criterion for VMEs for the *Enterobacteriaceae* strains (1.1% [1/92 isolates]).

The rate of clinical isolate nonsusceptibility to ERV determined by ETEST for *Enterococcus* was 7.5% according to the FDA breakpoints. Overall performance of ETEST ERV with all clinical and challenge isolates of both *Enterococcus* species was 100.0% EA, 94.9% CA, VME rate of 33.3%, and ME rate of 3.1%. The adjusted VME and ME rates were 0.0% (Fig. 3 and Table 2).

### (ii) ETEST ERV performance with EUCAST breakpoints and ISO requirements for E. coli, K. pneumoniae, and *Enterococcus* isolates.

According to the EUCAST breakpoints, a total of 10 Escherichia coli and *Enterococcus* isolates (3.0%) were resistant to ERV by BMD. Among the indicated species approved by the EMA, EA was 99.3% (429/432 isolates). CA was established only for E. coli, E. faecalis, and E. faecium, because EUCAST did not set breakpoints for K. pneumoniae; the result was 100.0% (328/328 isolates) (Table 3).

**TABLE 3:** Clinical and challenge performance of ETEST ERV with EUCAST breakpoints

Organism	Total no.	No. within EA	EA (%)	Total no. evaluable	No. evaluable within EA	No. within CA	CA (%)	No. resistant	No. of VMEs	No. of MEs
Challenge set										
E. faecalis	14	14	100.0	14	14	14	100.0	1	0	0
E. faecium	3	3	100.0	3	3	3	100.0	0	NA^*a*^	0
E. coli	11	11	100.0	11	11	11	100.0	1	0	0
K. pneumoniae	14	14	100.0	14	14	NA	NA	NA	NA	NA
Subtotal	42	42	100.0	42	42	28	100.0	2	0	0
Clinical set										
E. faecalis	60	60	100.0	60	60	60	100.0	0	NA	0
E. faecium	60	60	100.0	60	60	60	100.0	2	0	0
E. coli	180	178	98.9	180	178	180	100.0	6	0	0
K. pneumoniae	90	89	98.9	90	89	NA	NA	NA	NA	NA
Subtotal	390	387	99.2	390	387	300	100.0	8	0	0
Combined set, total	432	429	99.3	432	429	328	100.0	10	0	0

a NA, not applicable.

### Trend analysis.

Trend analyses were calculated for all species together and for any individual species following FDA guidance. A trend to overestimate ETEST ERV MIC values, compared to the CLSI reference BMD method, was observed for C. freundii (38.57% [95% confidence interval [CI], 25.28% to 50.57%]), E. coli (37.70% [95% CI, 29.65% to 45.22%]), and K. aerogenes (50.00% [95% CI, 30.43% to 66.37%]). Of these species, only C. freundii reported categorical errors (7.0% were MEs [4/57 errors]), all of which were within EA with the reference method. No trend was observed for all species together or all other species.

## DISCUSSION

Here, we evaluated the performance of ETEST ERV, compared to the BMD reference method, according to FDA and EUCAST breakpoints for *Enterobacteriaceae* and *Enterococcus* isolates. Overall, ETEST ERV exceeded FDA and ISO performance criteria, demonstrating 99.6% and 99.3% EA and 97.3% and 100.0% CA for clinical and challenge isolates overall, respectively. A single, nonreproducible adjusted VME and 6 MEs from the *Enterobacteriaceae* family and 4 MEs from the *Enterococcaceae* family occurred with the FDA breakpoints. The clinical performance of the ETEST ERV test was found useful for determining the ERV MICs for both *Enterobacteriaceae* and enterococci, including those that produce ESBLs or carbapenemases and VRE. Among the 600 clinical isolates tested, including a part of the population selected for its resistance, the overall susceptibility to ERV according to the reference method was 87.0%. The mode MIC was low, at 0.25 μg/mL for *Enterobacteriaceae* and 0.032 μg/mL for enterococci (susceptible).

Strengths of the current study are the inclusion of isolates with different mechanisms of resistance to different β-lactam, vancomycin, and tetracycline antibiotics, the large number of clinical isolates (*n* = 600) included, the study design involving study sites in the United States and Europe, and the use of a CLSI/ISO BMD reference method with a standardized and validated preparation of MIC panels. A limitation of the current study is the lack of nonsusceptible C. koseri isolates. The lack of an intermediate interpretive category led to results obtained with E. cloacae, K. pneumoniae, E. faecium, and E. faecalis showing potential for MEs and/or VMEs, compared to the reference method. Also, footnotes were added to the package inserts describing the VME rate for *Enterobacteriaceae* and *Enterococcus* before and after adjustment and explaining that one VME was not within EA with the reference method when testing Klebsiella pneumoniae isolates. The trend to overestimate ETEST ERV MIC values for C. freundii, E. coli, and K. aerogenes, compared to the reference method, is also noted above. Some limitations and footnotes were required by the FDA mainly because of the VME rate before adjustment, despite the lack of underestimation trend and the implicitly recognized normal variability of AST methods at one dilution. It can be extremely difficult in such situation to avoid limitations when there is a unique breakpoint without any intermediate category.

This is the first study comparing the performance of this new ETEST ERV strip to that of the standard BMD method in a clinical setting. Overall, we report that the ETEST ERV demonstrated acceptable performance for *Enterobacteriaceae* and *Enterococcus* spp., compared to the reference BMD method. The results of this trial support FDA clearance and CE marking of ETEST ERV.
